# Evaluation of TUBEX-TF and OnSite Typhoid IgG/IgM Combo rapid tests to detect *Salmonella enterica* serovar Typhi infection during a typhoid outbreak in Harare, Zimbabwe

**DOI:** 10.1186/s13104-015-1015-1

**Published:** 2015-02-24

**Authors:** Andrew Tarupiwa, Simba Tapera, Sekesai Mtapuri-Zinyowera, Peter Gumbo, Vurayai Ruhanya, Muchaneta Gudza-Mugabe, Ngoni Xmas Majuru, Nyasha Chin’ombe

**Affiliations:** National Microbiology Reference Laboratory, P O Box ST 749, Southerton, Harare Zimbabwe; Department of Medical Microbiology, College of Health Sciences, University of Zimbabwe, P.O. Box A178, Avondale, Zimbabwe

**Keywords:** Typhoid fever, *Salmonella*, Rapid Tests, Evaluation

## Abstract

**Background:**

*Salmonella enterica* serovar Typhi, the causative agent of typhoid, is endemic in most parts of the world especially in Africa. Reliable and rapid diagnosis of the bacterium is therefore critical for confirmation of all suspected typhoid cases. In many parts of Zimbabwe, laboratory capacity to isolate the microorganism by culture method as a way of diagnosis has limitations. In this study, two rapid serological kits, TUBEX-TF and OnSite Typhoid IgG/IgM Combo, were evaluated for possible expeditious diagnosis of *Salmonella enterica* serovar Typhi infection during a typhoid outbreak in Zimbabwe.

**Methods:**

Blood was collected from patients with clinical signs and symptoms of typhoid in Harare, Zimbabwe during an outbreak. The standard culture method was used to diagnose the disease. Two rapid kits, the TUBEX-TF and OnSite Typhoid IgG/IgM Combo, were also used in parallel to diagnose typhoid according to manufacturers’ instructions. The diagnostic accuracy of the two kits was evaluated using the culture method as the gold standard.

**Results:**

From all the cases diagnosed by the blood culture (n = 136), we enrolled 131 patients for the TUBEX-TF and 136 for the OnSite Typhoid IgG/IgM Combo tests. With the culture method as a reference standard, we found that TUBEX-TF test was 100% sensitive and 94.12% specific, with 63.16% positive and 100% negative predictive values (NPVs) and the OnSite Typhoid IgG/IgM Combo test was 100% sensitive and 94.35% specific, with 63.16% positive and 100% NPVs.

**Conclusion:**

Our results indicated that TUBEX-TF and OnSite Typhoid IgG/IgM Combo rapid tests were useful tools for the rapid diagnosis of *Salmonella enterica* serovar Typhi infection during typhoid outbreaks in Zimbabwe. The tests performed very well in laboratory evaluations of blood culture-confirmed typhoid cases in Harare, Zimbabwe.

## Background

Typhoid fever is an important acute disease that is caused by *Salmonella enterica* serovar Typhi infection [1]. It is normally associated with prolonged fever, headache, malaise and bowel disturbances in both adults and children [2]. In some cases, the disease can cause fatal complications such as intestinal perforations, gastrointestinal hemorrhages, encephalitis and cranial neuritis [2,3]. Annually, *Salmonella enterica* serovar Typhi is estimated to cause about 22 million cases of typhoid fever and 200 000 – 600 000 deaths globally [4,5]. The actual burden of the disease is however underestimated especially in most developing countries where most cases go undiagnosed. Laboratory detection of *Salmonella enterica* serovar Typhi is therefore crucial for confirmation of suspected typhoid cases [1]. Available diagnostic methods for the diagnosis of the bacterium include culture of the microorganism from patient samples, serological tests and nucleic acid-based methods. Although the culture method is currently the gold standard for diagnosis of *Salmonella enterica* serovar Typhi infection, its turnaround time is long (2–3 days) and it requires laboratory infrastructure that is not affordable to many resource-limited settings. However, all other test methods are normally evaluated against the culture method to ensure their appropriateness, efficiency and effectiveness. Serological tests such as hemagglutination test and enzyme-linked immunosorbent assay (ELISA) have also been used to diagnose *Salmonella enterica* serovar Typhi infection, but they are limited in specificity and sensitivity [6]. The Widal test, which measures agglutinating antibodies against the *Salmonella enterica* serovar Typhi H and O antigens, was the diagnostic standard for many years, but is no longer widely used in most parts of the world due to poor sensitivity and specificity [7]. Commercial rapid antibody tests that detect the presence of *Salmonella enterica* serovar Typhi antigens/antibodies have been made available in recent years and are now used in early outbreak detection, patient management and surveillance [1,8]. They can potentially be used as point-of-care (POC) tests and they are rapid and user-friendly. They also do not require specialized laboratory or well-trained staff. The clinical utility of some of these rapid tests has not been evaluated in most African countries where typhoid fever is endemic. The objective of this study was to evaluate two commercially available rapid diagnostic kits, the TUBEX-TF and the OnSite Typhoid IgG/IgM Combo for the laboratory detection of *Salmonella enterica* serovar Typhi infection in Zimbabwe.

## Methods

### Ethical approval and informed consent

Institutional and ethical permission to carry out the study was obtained from the Ministry of Health and Child Welfare (MOHCW) of Zimbabwe and the City of Harare Health Services Department (CHHSD). The study constituted public health response and program evaluation to typhoid outbreak. Adult participants and parents/guardians of sick children provided verbal informed consent before blood samples were collected.

### Patients and specimen collection

The study was carried out in Harare at National Microbiology Reference Laboratory and blood specimens were collected from the Beatrice Road Infectious Disease Hospital laboratory using samples from patients seeking treatment at Kuwadzana Polyclinic (Harare) during an outbreak of typhoid from March 2012 to June 2012. Specimens were collected from patients who presented with typical signs and symptoms of typhoid. Patients (adults) gave 10 ml of blood or 3 ml from children (3–5 yrs old) upon routine venipuncture. All patients had no history of treatment with antibiotics or any other medications.

### Laboratory test methods

Blood samples collected from the patients were screened for *Salmonella enterica* serovar Typhi using standard laboratory protocol for blood culture method. Briefly, 5 ml or 3 ml of fresh blood was used to inoculate into blood culture bottles, each containing 45 ml of broth. The blood cultures were incubated at 37°C for 24 hours. Cultures which were negative after 24 hours were further incubated for 7 days. Positive samples were plated on MacConkey agar, Chocolate and Blood agar and incubated at 37°C for 24 hours. Plates were examined for *Salmonella enterica* serovar Typhi colonies.” Standard biochemical and serological tests were performed to confirm the organism. Serological testing was done using slide agglutination with the following antisera for confirmation: *Salmonella* O factor A-S, monofacter 09, 12, VI, *Salmonella* H: Polyvalent Phase1 and 2 (a-z29), and *Salmonella* H monofacter d (*Salmonella enterica* serovar Typhi reacted positively with all the above).

The bleeds were also used for parallel evaluation with two typhoid rapid tests, the TUBEX-TF and the OnSite Typhoid IgG/IgM Combo tests. The rapid tests were carried out according to manufacturers’ instructions. The TUBEX® TF is a competitive immunoassay which detects the presence of anti-09*Salmonella* enterica serovar Typhi antibodies in the patient’s serum by assessing their ability to inhibit the reaction between the antigen and antibody in the reagents. The level of inhibition is proportional to the concentration of anti-09*Salmonella* antibodies in the sample. The method specifically detects IgM antibodies to the *Salmonella enterica* serovar Typhi 09 lipopolysaccharide antigen. The OnSite Typhoid IgG/IgM Combo Rapid Test is a lateral flow chromatographic immunoassay and the test cassette consists of a burgundy coloured conjugate pad containing recombinant *Salmonella enterica* serovar Typhi H antigen and O antigen conjugated with colloid gold (Typhoid conjugates) and rabbit IgG-gold conjugates. There is also a nitrocellulose membrane strip containing two test bands (M and G bands) and a control band (C band). The M band is pre-coated with monoclonal anti-human IgM for the detection of IgM anti- *Salmonella enterica* serovar Typhi.

### Evaluation of diagnostic accuracy of the rapid kits

The results from the two rapid kits were evaluated against blood culture method as the gold standard. For each of the two tests, sensitivity, specificity, positive and negative predictive values were calculated using standard formulae. Sensitivity, which is the percentage of positive individuals correctly identified as such, was calculated using the formula: Sensitivity = number of true positives (TP)/(number of TP + number of false negatives (FN)) × 100%. Specificity, which is the percentage of negative individuals correctly identified as such, was calculated with the formula: Specificity = number of true negatives (TN)/(number of TN + number of false positives (FP)) × 100%. Positive predictive value (PPV), the proportion of positive test results that are truly positive, was calculated using the formula: PPV = TP/(TP + FP) × 100%, and the negative predictive value (NPV), which is the proportion of negative test results that are truly negative was calculated using the formula: NPV = TN/(TN + FN) × 100%.

## Results

### Patient data

Patients in this study had symptoms and signs of typhoid, which included persistent and high fever (>38°C oral temperature), chills malaise, headache, sore throat, cough and sometimes abdominal pain and constipation or diarrhea. A total number of 131 and 136 patients were analyzed using the TUBEX-TF and OnSite Typhoid IgG/IgM Combo kits respectively.

### Rapid test results

The diagnostic relevance of the two rapid antibody tests for typhoid was evaluated by calculation the sensitivity, specificity and the positive predictive values. The results are summarized in Table 1. With the culture method as a reference standard, it was found that TUBEX-TF test was 100% sensitive and 94.12% specific, with 63.16% positive and 100% negative predictive values (NPVs) and the OnSite Typhoid IgG/IgM Combo test was 100% sensitive and 94.35% specific, with 63.16% positive and 100% NPVs (Table 1).

**Table 1:** **Diagnostic accuracy of the TUBEX-TF and OnSite Typhoid IgG/IgM Combo tests with culture as the gold standard**

**Test**	**Sensitivity (%)**	**Specificity (%)**	**PPV (%)**	**NPV (%)**
TUBEX-TF (n = 131)	100	94.12	63.16	100
OnSite Typhoid IgG/IgM Combo (n = 136)	100	94.34	63.16	100

## Discussion

Diagnostic tests always need to be evaluated to establish whether their performance is adequate for their intended purpose. They should also be evaluated under the conditions they are most likely to be used and in a particular human population or setting. In this study, we evaluated the diagnostic accuracy of two rapid antibody tests, TUBEX-TF and OnSite Typhoid IgG/IgM Combo, during a typhoid fever outbreak in Harare in Zimbabwe. The two kits evaluated demonstrated a high degree of sensitivity and a reasonably high degree of specificity (Table 1). The PPVs for both kits were 63% and the NPVs were 100%. The PPV figures were low suggesting that the kits were not doing as good as the gold standard. However since PPV decreases with decrease in prevalence, the low prevalence of typhoid in the population could be the cause of the perceived low figures.

The two rapid tests have also been evaluated elsewhere and gave varying results. A study in Bangladesh evaluated the TUBEX-TF rapid test in which blood culture was used as a reference test [9]. The test was only 60% sensitive and 58% specific, with 90% positive and 58% negative predictive values [9]. In this study, the diagnostic value of the TUBEX-TF test was shown to be poor. In another study in Papua New Guinea, the TUBEX-TF test was demonstrated to have sensitivity and specificity of 77.3% and 87.4%, respectively when culture was used as a gold standard [10]. The PPV and NPV were 31% and 95% respectively [10]. When a composite gold standard of blood culture and PCR was used, the sensitivity and specificity were 51.1% and 88.3%, with 31% positive and 95% negative predictive values [10]. Using both molecular and culture methods can therefore help to improve the evaluation of rapid tests in detection of *Salmonella enterica* serovar Typhi infection [6,10]. The TUBEX-TF test was also shown to perform badly in the Philippines and Vietnam [11,12]. However, a study on typhoid cases from Hong Kong and Malaysia demonstrated 100% sensitivity and 100% specificity by the TUBEX-TF test [13]. In Africa, the TUBEX-TF test performed poorly in studies in South Africa, Tanzania and Egypt [14,15]. The OnSite Typhoid IgG/IgM test has also been previously evaluated. When a total of 234 samples from susceptible subjects were evaluated by the OnSite Typhoid IgG/IgM Combo Rapid Test and with a commercial *Salmonella enterica* serovar Typhi IgM EIA as a standard, sensitivity and specificity were 91.1% and 99.0% respectively [16]. The possible reason why in our study the two rapid tests performed well was because that the study was carried out during a typhoid outbreak. This evaluation of two rapid tests could be of great importance in Zimbabwe where we have sporadic typhoid outbreaks that need to be confirmed routinely. The kits also demonstrated performance features which were acceptable. The tests were easy to use, robust and could be stored at temperatures ranging from refrigeration to room temperature. They could easily be used outside the laboratory as point-of-care tests. Further evaluations of the two rapid tests are needed when there is no outbreak of the typhoid disease in Zimbabwe. Future evaluations of such rapid tests should also use molecular tests such as polymerase chain reaction as reference standard since they more sensitive and specific than culture.

## Conclusions

This study produced promising results on the diagnostic performance of two rapid typhoid tests, TUBEX-TF and OnSite Typhoid IgG/IgM Combo in detecting typhoid fever cases in Harare, Zimbabwe. The rapid tests can be useful tools in the diagnosis of *Salmonella enterica* serovar Typhi during typhoid outbreaks in Zimbabwe.
